# Low back pain in healthy postmenopausal women and the effect of physical activity: A secondary analysis in a randomized trial

**DOI:** 10.1371/journal.pone.0177370

**Published:** 2017-05-10

**Authors:** Mirca Marini, Benedetta Bendinelli, Melania Assedi, Daniela Occhini, Maria Castaldo, Jacopo Fabiano, Marco Petranelli, Mario Migliolo, Marco Monaci, Giovanna Masala

**Affiliations:** 1 Department of Experimental and Clinical Medicine, Section of Anatomy and Histology, University of Florence, Florence, Italy; 2 Cancer Risk Factors and Lifestyle Epidemiology, Cancer Research and Prevention Institute (ISPO), Florence, Italy; 3 Department of Experimental and Clinical Medicine, University of Florence, Florence, Italy; 4 President of the Florentine Sports Medicine Association (FMSI – CONI), Florence, Italy; Pennington Biomedical Research Center, UNITED STATES

## Abstract

Epidemiological studies on the prevalence of musculoskeletal pain have consistently shown that this is a relevant health problem, with non-specific low back pain (LBP) being the most commonly reported in adult females. Conflicting data on the association between LBP symptoms and physical activity (PA) have been reported. Here, we investigated the prevalence of LBP and the effect of a 24-month non-specific PA intervention on changes in LBP prevalence in a series of Italian healthy postmenopausal women. We performed a secondary analysis in the frame of the DAMA trial, a factorial randomized intervention trial aimed to evaluate the ability of a 24-month intervention, based on moderate-intensity PA, and/or dietary modification, in reducing mammographic breast density in healthy postmenopausal women. The PA intervention included at least 1 hour/day of moderate PA and a more strenuous weekly activity, collective walks and theoretical group sessions. A self-administered pain questionnaire was administered at baseline and at the end of the intervention. The questionnaire was specifically structured to investigate the occurrence of musculoskeletal pain, the body localization, intensity and duration of the pain. Two hundred and ten women (102 randomized to PA intervention, 108 not receiving the PA intervention) filled out the questionnaires. At baseline LBP was present in 32.9% of the participants. Among women randomized to the PA intervention, LBP prevalence at follow up (21.6%) was lower than at baseline (33.3%) (p = 0.02), while in women who did not receive the PA intervention the LBP prevalence at baseline and follow up were 32.4% and 25.9%, respectively (p = 0.30). Overall, there was no significant between-group effect of PA intervention on LBP. Further studies are needed to understand the role of non-specific PA intervention, aimed to improve overall fitness, on LBP prevalence.

## Introduction

Epidemiological studies have reported a high prevalence of musculoskeletal pain in the adult general population, particularly in women [1, 2]. Among spinal musculoskeletal disorders, low back pain (LBP) is an extremely common problem that most people, mainly females, experience at some point during their life [3]. In particular, an increasing prevalence of LBP with a peak in the sixth decade has been reported [4]. LBP is a major health problem throughout the world causing considerable physical and psychological impairments, absence from work and high socioeconomic costs [4, 5].

LBP is usually defined as pain localized below the margin of the last ribs (costal margin) and above the inferior gluteal line, with or without lower limb pain. LBP is typically classified as “specific” or “non-specific” [6, 7]. Non-specific LBP is characterized by the absence of structural anatomical changes [7] and seems to affect more than 85% of individuals [5, 7]. The etiology of LBP is multi-factorial and not fully understood. Previous studies have identified several risk factors for LBP including age, female gender, educational status, obesity, smoking, sleep deprivation, prolonged driving, computer usage and lack of exercise. In particular, it has been suggested that physical deconditioning may play an important role in the etiology of chronic LBP. Subjects practising strenuous physical activities and subjects with sedentary lifestyle are both at increased risk for the development of chronic LBP [8]. Psychosocial factors, such as stress and depression, may also play a role in this scenario [4].

There are several treatments for LBP (e.g., medications, physical intervention), but their efficacy is not fully proven [7]. Physical activity (PA) has been suggested as an effective treatment for patients with sub-acute or chronic non-specific LBP [9], though which types of specific exercises may be most beneficial is still unclear. To prevent chronic pain, it is very important that the treatment focuses on promotion of PA despite the pain. Therefore, in the acute phase educational and PA promoting measures should be the primary treatment options [5]. However, future strategies should focus on possible interventions aimed at preventing new episodes of LBP with greater impact both on the health status and the reduction of socio-economic costs.

In this context, the objective of our study was to investigate, in a series of Italian healthy postmenopausal women, the prevalence of LBP, and the effect of a 24-month PA intervention on possible changes in LBP prevalence.

## Materials and methods

### Study participants

The present study represents a secondary analysis of the DAMA trial [10] and includes the 210 participants, out of the 234 overall women enrolled in the DAMA trial, who also answered a questionnaire on non-specific musculoskeletal pain both at baseline and after 24-month intervention. Due to logistic and organizational reasons the remaining 24 participants did not complete the baseline and/or follow up questionnaires on non-specific musculoskeletal pain and were excluded from the current analyses.

The DAMA (Diet, physical Activity and MAmmography) trial (ISRCTN28492718) is a factorial randomised trial aimed to evaluate the ability of a 24-month intervention, based on moderate-intensity PA, and/or dietary modification, in reducing mammographic breast density (MBD) in healthy postmenopausal women. The DAMA trial was approved by the Ethics Committee of the Local Health Authority in Florence (Italy). Informed consent form was signed by all study participants.

Methods and design of the DAMA trial have been previously described in detail [10]. Briefly a total of 234 Italian healthy postmenopausal women, aged 50–69 years, not using Hormone Replacement Therapy, non-smoking and with high MBD, a well-known risk factor for breast cancer (>50% as assessed in the frame of the local screening programme), were recruited to participate at the trial. At baseline and at the end of the DAMA intervention, all participants filled out a food frequency questionnaire (FFQ) to assess dietary habits and a lifestyle questionnaire to assess lifestyle behaviours including occupational, household and recreational PA [11]. Anthropometric parameters, such as height and weight, were also measured using standard protocols and used to calculate body mass index (BMI, kg/m^2^). After the baseline visit women were randomly assigned to one of the following four study arms: 1) “dietary intervention” in which women received a series of specific practical and educational activities on healthy dietary habits; 2) “PA intervention” in which women received a series of specific practical and educational activities on healthy PA habits; 3) “dietary and PA intervention” in which women received exactly the same activities proposed in arm 1 and 2; and 4) in which women received only general advices on both dietary and PA healthy habits. This factorial design allows to evaluate separately the effect of dietary or PA intervention comparing the groups receiving the same treatment (in this analysis the PA intervention) with the groups not receiving the specific treatment.

### Physical activity intervention

According to the DAMA trial protocol, the participants included in the present study and randomized to the arms receiving the PA intervention (study arms 2 and 3; 108 women), hereafter referred to as “the PA intervention group”, were required to daily increase moderate recreational activities up to 1 hour/day (corresponding to about 3–5.9 metabolic equivalent (MET)-hours/day), in combination with more strenuous weekly activity (6–10 MET-hours/week) and to participate in theoretical group sessions. The exercise program was planned by an exercise specialist and was applied gradually according to the baseline level of activity of each subject. Suggested moderate activities were, for example, walking, biking and slow dancing. Women were also provided with some equipment (an elastic band, dumbbells, a gym mat) and a specific booklet to exercise at home. In addition, women were requested to attend weekly 1-hour exercise sessions led by exercise specialists in an appropriate fitness facility for the whole study period (total 97 sessions). The primary aim of these exercise sessions was to increase aerobic capacity, physical strength, postural control, coordination and mobility of the limbs and trunk. The PA intervention protocol also included participation in 6 collective walks and 6 theoretical group sessions (approximately 25 women/session) in which evidences about PA health benefits were presented and discussed and women were instructed on how to gradually increase daily levels of PA, breathe properly during exercise, improve and keep good posture. Moreover, women were requested to keep 5 periodical weekly written PA diaries in order to monitor the achievement and maintenance of the intervention aims.

Women randomized to the arms not receiving the PA intervention (study arms 1 and 4; 102 women), hereafter referred to as “the control group”, received, at baseline, only a leaflet with general advices on healthy PA patterns and were invited to participate in a single specific conference on the general beneficial effects of PA.

### Pain questionnaire

A specific self-administered questionnaire on non-specific musculoskeletal pain was filled, at baseline and at the end of the 24 months intervention, by participants. The pain questionnaire included questions elaborated through translation and adaptation from previously validated instruments used in the literature [12–14].

Specifically, women were asked if they had recently experienced pain in any body areas and when the last episode occurred (response option were: last 48 hours, last 1–2 weeks, last 3–4 weeks, more than 3 months ago). The specific localization of the pain was then requested (neck, shoulders, elbows, hip, knee, leg, cervical spine, thoracic spine, lumbar spine, sacral spine). One or more pain localizations could be specified by each participant. Information on pain localization was then combined to represent the following anatomical sites: upper back (i.e. neck and cervical spine), lower limb (i.e. knee and leg), mid back (i.e. thoracic spine), low back (i.e. lumbar spine and sacral spine) and spine (i.e. neck, cervical spine, thoracic spine, lumbar spine and sacral spine).

Study participants were also requested to indicate the pain intensity of the most recent episode in each body localization according to a numeric rating scale (NRS) [15]. The NRS for pain is an unidimensional measure of pain intensity ranging from 0 (no pain) to 10 (pain as bad as you can imagine/worst pain imaginable). Pain intensity, assessed by NRS scale, was categorized into three levels: score 1–3 = mild, score 4–6 = moderate, and score ≥7 = severe pain [16].

To assess the pain duration of the most recent episode, the following question was asked: “*How long have you had your current pain problem*?”. The response options were: 0 days; 1–2 days; 3–7 days; 8–14 days; 15–30 days; 1 month; 2 months; 3–6 months; 6–12 months; over 1 year.

In addition, the subjects were asked to report the pain frequency during the last 12 months: 0 time; 1 time, 2 times, 3 times, 4 times; over 4 times.

### Statistical analysis

The current analysis represents a secondary analysis focused on postmenopausal women participating into the DAMA trial who completed the pain questionnaire at baseline and at follow up. The primary outcome of the present study was the prevalence of LBP.

Assuming an expected prevalence of LBP of 40% at baseline [3, 17] and a LBP prevalence of 20% in the PA intervention group after the 24-month intervention and with a sample size of 210 study participants (108 women in the PA intervention group and 102 women in the control group), the statistical power for our analyses was 81%. Distribution of the main baseline characteristics was investigated overall and according to PA intervention. For continuous variables means (standard deviations), medians (10°- 90° percentiles) and *p values* from mean comparison test between groups (PA intervention Yes/No) were calculated. For categorical variables frequency number, relative percentages and *p values* from Fisher exact test were calculated. Prevalence and body localization of pain at baseline were also investigated. Specific analyses were carried out for LBP prevalence at baseline and follow up. Among women reporting LBP at baseline, pain intensity and duration were evaluated.

The proportion of women reporting LBP presence before and after the 24-month intervention was investigated according to PA intervention and following the intention to treat principle. The McNemar test for matched pairs was performed in order to evaluate the differences in LBP presence at baseline and follow-up within groups. The difference in LBP prevalence at follow up according to PA intervention was evaluated and a test of proportion was performed. A crude and a multivariate logistic model (adjusted for age at enrolment, body weight, educational level, total PA level and LBP presence/absence at baseline) were also run in order to evaluate the real effect of the PA intervention on LBP prevalence.

## Results

Overall, 210 healthy postmenopausal women, aged 50–69 years, were involved in this study. Baseline characteristics of the participants are detailed in Table 1. The PA intervention group included 102 women and the control group included 108 women. No significant differences between the two groups at baseline were observed for age, BMI, educational level, leisure-time PA and occupational activities (Table 1).

**Table 1:** Baseline characteristics of the study participants overall and according to physical activity (PA) intervention.

Baseline	Total (N = 210)	PA intervention		p value *
		Yes (N = 102)	No (N = 108)	
*Mean (SD)*				
*Median (10°-90° percentiles)*				
**Age (years)**	59.0 (5.1) 58.3 (52.4–66.4)	59.3 (4.7) 59.0 (53.4–65.6)	58.7 (5.4) 57.5 (51.9–66.9)	0.37
**Body mass index (kg/m**^**2**^**)**	24.3 (3.4) 23.8 (20.3–28.8)	24.5(3.5) 23.7 (20.7–28.8)	24.2 (3.3) 23.8 (20.1–28.6)	0.56
**Non occupational physical activity (h/week)**	26.8 (14.3) 24.2 (11.8–46.5)	26.7 (15.3) 22.4 (11.7–52.0)	26.9 (13.3) 26.0 (11.9–45.2)	0.90
- Recreational physical activity (h/week)	6.4 (4.5) 5.5 (1.5–12.4)	6.1 (4.2) 5.0 (1.2–12.0)	6.7 (4.7) 6.0 (1.5–13.0)	0.31
- Household physical activity (h/week)	20.4 (13.2) 14.6 (5.2–40.1)	20.6 (14.2) 14.1 (6.3–40.7)	20.2 (12.2) 15.4 (4.4–38.5)	0.83
*N (%)*				
**Occupational physical activity**				
- Sedentary	74 (35.2)	35 (34.3)	39 (36.1)	
- Standing	29 (13.8)	13 (12.7)	16 (14.8)	
- Manual	15 (7.1)	7 (6.9)	8 (7.4)	
- Heavy manual	2 (0.9)	1 (1.0)	1 (0.9)	
- No paid work	90 (42.9)	46 (45.1)	44 (40.7)	0.96
**Level of education**				
- None/primary school	59 (28.1)	25 (24.5)	34 (31.5)	
- High school	90 (42.9)	44 (43.1)	46 (42.6)	
- University	61 (29.0)	33 (32.3)	28 (25.9)	0.45

* P values from mean comparison test for continuous variables and from Fisher exact test for categorical variables.

**Fig 1:**
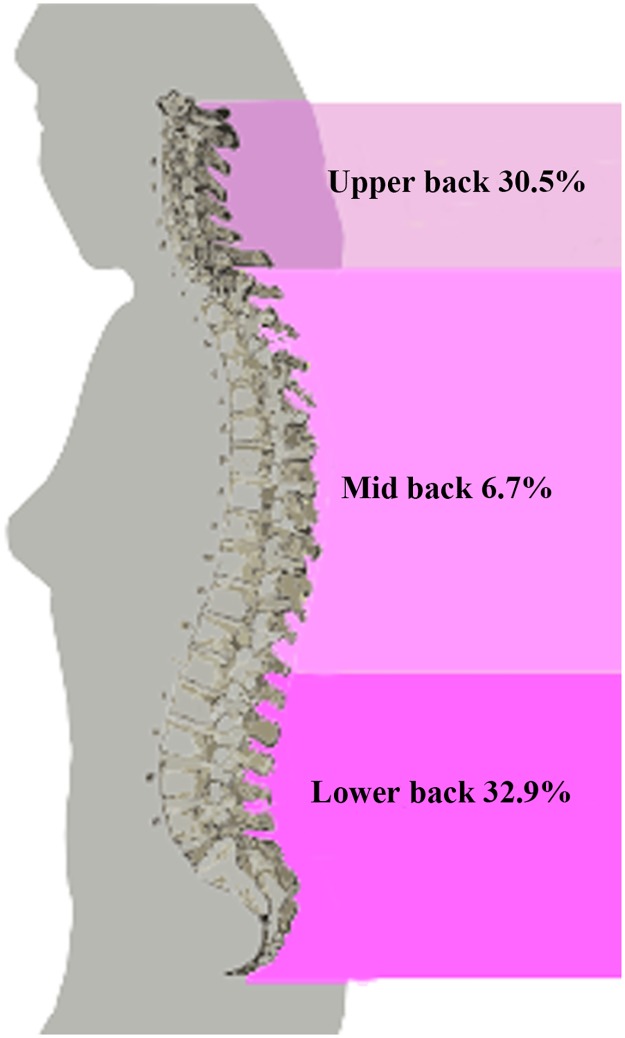
Baseline prevalence (percentage) of different types of back pain in the 210 study women.

At baseline, 56 women (26.6%) had no pain while 154 (73.3%) reported pain referred to one or more different parts of the body. The main localization of pain was the spine (55.2%), mostly in the low back area (32.9%), followed by lower limb (21.9%), shoulder (21.4%), hip (9.0%) and elbow (5.7%) (Table 2). In particular, the prevalence of LBP (32.9%) was higher than that of upper and mid back pain (30.5% and 6.7%, respectively) (Fig 1).

**Table 2:** Prevalence and body localization of pain reported by study women at baseline, overall and according to physical activity (PA) intervention.

Baseline	Total *N (%)*	PA intervention		p value *
		Yes *N (%)*	No *N (%)*	
**Pain presence**	**(N = 210)**	**(N = 102)**	**(N = 108)**	
Yes	154 (73.3)	74 (72.5)	80 (74.1)	
No	56 (26.6)	28 (27.5)	28 (25.9)	0.80
**Body localization** ^a^				
Spine	116 (55.2)	58 (56.9)	58 (53.7)	0.65
- Upper back	64 (30.5)	34 (33.3)	30 (27.8)	0.38
- Mid back	14 (6.7)	6 (5.9)	8 (7.4)	0.66
- Low back	69 (32.9)	34 (33.3)	35 (32.4)	0.89
Shoulder	45 (21.4)	21 (20.6)	24 (22.2)	0.77
Elbow	12 (5.7)	2 (2.0)	10 (9.3)	0.02
Hip	19 (9.0)	7 (6.9)	12 (11.1)	0.28
Lower limb	46 (21.9)	23 (22.5)	23 (21.3)	0.83

* *P* values from Chi squared test

^a^ One or more pain localizations could be reported by each participant.

As regard the 69 women reporting LBP at baseline, 78.3% (54 out of 69) referred a pain duration lower than 3 months (acute pain). LBP was defined as severe in 24 (34.8%), moderate in 28 (40.6%) and mild in 17 (24.6%) women. Moreover, 44.4% of women reporting LBP with duration lower than 3 months referred five pain episodes or more in the last year.

No differences in LBP prevalence emerged at baseline between the PA intervention group (33.3%) and the control group (32.4%, p = 0.89).

Table 3 shows the overall prevalence of self-reported LBP at baseline and after the 24-month intervention by group. In the PA intervention group LBP prevalence at follow up (21.6%) was lower than at baseline (33.3%) (McNemar test p = 0.02). Within this group 18 women (17.6%) reporting LBP at baseline did not refer LBP at follow up, while 6 (5.9%) without LBP at baseline reported LBP at follow up. In the control group the reported LBP prevalence at baseline and follow up were 32.4% and 25.9%, respectively (McNemar test p = 0.30). Within this group 20 (18.5%) out of the women reporting LBP at baseline did not refer LBP at follow up, while 13 (12.0%) without LBP at baseline reported LBP at follow up.

**Table 3:** Distribution, N (%), of low back pain presence (yes/no) at baseline and follow-up in the 210 study women according to physical activity (PA) intervention.

		PA intervention					
		Yes (N = 102)			No (108)		
		Follow-up					
		No pain	Yes pain	Total	No pain	Yes pain	Total
	**No pain**	62 (60.8)	6 (5.9)	68 (66.7)	60 (55.6)	13 (12.0)	73 (67.6)
**Baseline**	**Yes pain**	18 (17.6)	16 (15.7)	34 (33.3)	20 (18.5)	15 (13.9)	35 (32.4)
	**Total**	80 (78.4)	22 (21.6)	102	80 (74.0)	28 (25.9)	108
	**McNemar Test** ^a^	p = 0.02			p = 0.30		

^a^ P values from McNemar Test for paired data evaluating the differences in pain presence at baseline and follow-up within PA intervention group and control group.

When we compared the prevalence of reported LBP at follow up between the PA intervention group and the control group (21.6% and 25.9%, respectively), the difference (-4.3%; 95%CI -15.8%, 7.2%) did not reach the statistical significance (p = 0.46). In a multivariate analysis, a non significant inverse association between PA intervention and LBP presence at follow up emerged both in a crude model (OR 0.79; 95% CI 0.42–1.49) and in an adjusted model taking into account a series of confounders including the presence of LBP at baseline (OR 0.72; 95% CI 0.36–1.45).

## Discussion

In the present study, non-specific spinal pain showed a high prevalence in a large Italian series of healthy postmenopausal women, and LBP was more frequent than mid or upper back pain.

In particular, here we evaluated the effect of a 24-month non-specific PA intervention on LBP prevalence specifically in postmenopausal women, aged 50–69 years. Our McNemar test results revealed a significant lower prevalence of LBP at follow up than at baseline among women randomized to a PA intervention program combining supervised and non-supervised exercise sessions and theoretical education sessions. However, the enthusiasm that might be generated by this positive finding should be tempered by the observation that there was no significant between-group effect on LBP prevalence when comparing the PA intervention and control groups.

LBP prevalence in the general population has been investigated in numerous previous studies [4, 6, 7, 18]. LBP is very common, but its prevalence estimates vary possibly owing to differences in diagnostic criteria, LBP definitions and the population characteristics [19–21]. Otherwise there are few reports that described the association between LBP and PA [17]. Moreover, previous studies often included relatively shorter follow-up periods with great heterogeneity in gender and ages of participants [6] and the “dose” of PA was not well defined [22]. Therefore, lack of uniform definitions of both PA and LBP makes outcomes difficult to compare [22]. However, the most recent evidence suggests that exercise alone or in combination with education is effective in the prevention of LBP [23].

In this context, our present study has a specific focus on the prevalence of LBP in healthy postmenopausal women (aged 50–69 years) as well as the possible changes in LBP prevalence following a well-defined 24-month PA program, though non-specific for LBP. According to our data, the baseline prevalence of LBP was 32.8%. Our data are consistent with those described in previous studies [17, 24, 25]. In particular, in the MONICA study, LBP prevalence was 41% in a population consisting of 5798 subjects aged 25–79 years. Furthermore, LBP prevalence was higher among women (44.1%) than men and highest in the group aged 55–64 years [17]. Moreover, in that study women with LBP were more often smokers compared with women without LBP suggesting that smoking should be considered among the risk factors of LBP [17]. Of note, non-smoking habit was one of the eligibility criteria for women enrolled in the DAMA trial. Although the prevalence of LBP has been investigated in numerous studies [17], the present report is to our knowledge the first presenting data on non-specific LBP in a sample of Italian healthy post-menopausal women aged 50–69 years. Moreover, there are very few studies that described the association between LBP and levels of PA in adult [17, 26]. At variance with previous reports [17, 22], here we carefully described the methodology of a well planned PA intervention. In particular, in our protocol women were required to daily increase moderate recreational activities up to 1 hour/day, in combination with more strenuous weekly activity.

It is known that the non-specific LBP affects multiple aspects of an individual’s life including physical function with limitation of multiple activities, psychological well-being and the ability to work in the general adult population, and particularly among females [27–37]. In addition, fear of LBP recurrence may further limit activities [38]. As a consequence, LBP continues to be one of the most challenging issues in primary care [30].

In the light of the above, the identification of new effective and economic LBP prevention strategies with long-term impact appears of major importance [39]. Among non-pharmacological intervention, PA is widely recognized as an important health-related lifestyle factor with the potential of increasing the quality of life. The psychosocial and biological health benefits of PA are well established, and there is clear scientific evidence that regular and moderate PA can reduce the risk of morbidity of various diseases [40]. Of note, PA is also prescribed in rehabilitation programmes for LBP treatment [31, 41]. PA maintains or improves fitness helping to control the pain and decreasing the risk of acute pain in chronic LBP [41]. Current international guidelines advocate increasing PA as a management strategy for chronic LBP [42]. In particular, individually designed exercise programs delivered in a supervised format seem the most effective strategy [43]. PA may include either aerobic exercise or muscle strengthening and stretching exercises specific for the treatment area, even if currently there is limited evidence regarding the most effective type of exercise [38]. Patient recommendations for the treatment of acute LBP consist instead in the advice to stay active for maintaining fitness and flexibility [32]. Indeed, regular exercise seems the only effective strategy in prevention of LBP [6].

We can speculate that our non-specific PA intervention, primarily aimed to increase aerobic capacity, physical strength of all major muscle groups, postural control, coordination and mobility of the limbs and trunk, could improve functional ability maintaining and/or ameliorating the fitness level with consequent reduction in the prevalence of LBP at follow up. These aspects are crucial because acute LBP is often caused by trunk muscle weakness resulting from insufficient exercise, obesity, and improper posture [44]. Interestingly, the present PA intervention included not only practical but also educational activities aimed to either increase the overall fitness or reduce a sedentary behaviour possibly affecting the LBP prevalence. In this context, previous observations [37, 43] highlighted the importance of maintaining an active lifestyle through a well-defined and regular PA along with changing of sedentary lifestyle-related unhealthy behaviours to prevent non-specific LBP onset. However, our analyses revealed that there was no significant effect on LBP prevalence when comparing the PA intervention and control groups. Overall, these findings should be interpreted in the context of the limitations of our study. Indeed, the present data could be limited by the relatively small sample size investigated and a study design based on self-report assessments. In addition, it should be considered that this is a secondary analysis of the DAMA trial which was primarily designed to evaluate different outcomes. Therefore, the type of PA intervention was not specifically designed for LBP, but rather planned to improve the overall woman fitness. It is also possible that different results could be found when employing different exercise training modalities or studying other subject populations.

Despite the aforementioned limitations, this study has also some strengths worth mentioning. First, our study design provided a 24-month follow up time on healthy women from a randomized controlled clinical trial with a relatively high adherence rate. The reason for this might be because our PA intervention included also supervised training sessions which are important for adherence to study protocol. In particular, the employment of exercise professionals with knowledge of biomechanics and focusing on each subject’s needs are important to avoid PA interruption [7]. In addition, in our trial there were no adverse events related to the exercise intervention. Of note, this longitudinal study afforded an opportunity to investigate a randomly recruited female population, which was not selected on the basis of a previous history of LBP. Our findings also highlight the importance of the exercise maintenance as primary prevention against non-specific LBP development, thus supporting the need of health education through PA. Another strength of this study is that our PA protocol follows the current PA recommendations [45]. The present findings might therefore contribute to promote adherence to PA and provide a guide for educators and clinicians in LBP management. Finally, our data might even help in the design of further clinical trials in the same interventional area.

## References

[1] Bingefors K, Isacson D. Epidemiology, co-morbidity, and impact on health-related quality of life of self-reported headache and musculoskeletal pain—a gender perspective. Eur J Pain. 2004;8: 435–450. 10.1016/j.ejpain.2004.01.005 15324775

[2] Wijnhoven HA, de Vet HC, Picavet HS. Prevalence of musculoskeletal disorders is systematically higher in women than in men. Clin J Pain. 2006;22: 717–724. 10.1097/01.ajp.0000210912.95664.53 16988568

[3] Hoy D, Brooks P, Blyth F, Buchbinder R. The Epidemiology of low back pain. Best Pract Res Clin Rheumatol. 2010;24: 769–781. 10.1016/j.berh.2010.10.002 21665125

[4] Manchikanti L, Singh V, Falco FJ, Benyamin RM, Hirsch JA. Epidemiology of low back pain in adults. Neuromodulation. 2014;17: 3–10. 10.1111/ner.12018 25395111

[5] Bachmann S, Oesch P. Physiotherapy and rehabilitation for low back pain. Ther Umsch. 2013;70: 543–548. 10.1024/0040-5930/a000444 23985153

[6] van Tulder M, Koes B, Bombardier C. Low back pain. Best Pract Res Clin Rheumatol. 2002;16: 761–775. 12473272 10.1053/berh.2002.0267

[7] Lizier DT, Perez MV, Sakata RK. Exercises for treatment of nonspecific low back pain. Rev Bras Anestesiol. 2012;62: 838–846. 23176991 10.1016/S0034-7094(12)70183-6

[8] Notarnicola A, Fischetti F, Maccagnano G, Comes R, Tafuri S, Moretti B. Daily pilates exercise or inactivity for patients with low back pain: a clinical prospective observational study. Eur J Phys Rehabil Med. 2014;50: 59–66. 24104699

[9] Saner J, Kool J, Sieben JM, Luomajoki H, Bastiaenen CH, de Bie RA. A tailored exercise program versus general exercise for a subgroup of patients with low back pain and movement control impairment: A randomised controlled trial with one-year follow-up. Man Ther. 2015;20: 672–679. 10.1016/j.math.2015.02.005 25770419

[10] Masala G, Assedi M, Caini S, Ermini I, Occhini D, Castaldo M, et al The DAMA trial: a diet and physical activity intervention trial to reduce mammographic breast density in postmenopausal women in Tuscany, Italy. Study protocol and baseline characteristics. Tumori. 2014;100: 377–385. 10.1700/1636.17890 25296586

[11] Palli D, Berrino F, Vineis P, Tumino R, Panico S, Masala G, et al EPIC-Italy.A molecular epidemiology project on diet and cancer: the EPIC-Italy Prospective Study. Design and baseline characteristics of participants. Tumori. 2003;89: 586–593. 14870823 10.1177/030089160308900602

[12] Zelman DC, Gore M, Dukes E, Tai KS, Brandenburg N. Validation of a modified version of the brief pain inventory for painful diabetic peripheral neuropathy. J Pain Symptom Manage. 2005;29: 401–410. 10.1016/j.jpainsymman.2004.06.018 15857744

[13] Masiero S, Carraro E, Sarto D, Bonaldo L, Ferraro C. Healthcare service use in adolescents with non-specific musculoskeletal pain. Acta Paediatr. 2010;99: 1224–1228. 10.1111/j.1651-2227.2010.01770.x 20219047

[14] Bonezzi C, Nava A, Barbieri M, Bettaglio R, Demartini L, Miotti D, et al Validazione della versione italiana del Brief Pain Inventory nei pazienti con dolore cronico. MinervaAnestesiol. 2002; 68: 607–611. 12244292

[15] Hawker GA, Mian S, Kendzerska T, French M. Measures of adult pain: Visual Analog Scale for Pain (VAS Pain), Numeric Rating Scale for Pain (NRS Pain), McGill Pain Questionnaire (MPQ), Short-Form McGill Pain Questionnaire (SF-MPQ), Chronic Pain Grade Scale (CPGS), Short Form-36 Bodily Pain Scale (SF-36 BPS), and Measure of Intermittent and Constant Osteoarthritis Pain (ICOAP). Arthritis Care Res (Hoboken). 2011;63: S240–252. 22588748 10.1002/acr.20543

[16] Gong G, Li J, Li X, Mao J. Pain experiences and self-management strategies among middle-aged and older adults with arthritis. J Clin Nurs. 2013;22: 1857–1869. 10.1111/jocn.12134 23534697

[17] Björck-van Dijken C, Fjellman-Wiklund A, Hildingsson C. Low back pain, lifestyle factors and physical activity: a population based-study. J Rehabil Med. 2008;40: 864–869. 10.2340/16501977-0273 19242625

[18] Hoy D, Bain C, Williams G, March L, Brooks P, Blyth F, et al A systematic review of the global prevalence of low back pain. Arthritis Rheum. 2012;64: 2028–2037. 10.1002/art.34347 22231424

[19] Balagué F, Mannion AF, Pellisé F, Cedraschi C. Non-specific low back pain. Lancet. 2012;379: 482–491. 10.1016/S0140-6736(11)60610-7 21982256

[20] Juniper M, Le TK, Mladsi D. The epidemiology, economic burden, and pharmacological treatmen of chronic low back pain in France, Germany, Italy, Spain and the UK: a literature-based review. Expert Opin Pharmacother. 2009;10: 2581–2592. 10.1517/14656560903304063 19874246

[21] Edmond SL, Felson DT. Prevalence of back symptoms in elders. J Rheumatol. 2000;27: 220–225. 10648042

[22] Heneweer H, Vanhees L, Picavet HS. Physical activity and low back pain: a U-shaped relation? Pain. 2009;143: 21–25. 10.1016/j.pain.2008.12.033 19217208

[23] Steffens D, Maher CG, Pereira LS, Stevens ML, Oliveira VC, Chapple M, et al Prevention of low back pain: a systematic review and meta-analysis. JAMA Intern Med. 2016;176: 199–208. 10.1001/jamainternmed.2015.7431 26752509

[24] Waxman R, Tennant A, Helliwell P. A prospective follow-up study of low back pain in the community. Spine (Phila Pa 1976). 2000;25: 2085–2090. 10954640 10.1097/00007632-200008150-00013

[25] Gourmelen J, Chastang JF, Ozguler A, Lanoë JL, Ravaud JF, Leclerc A. Frequency of low back pain among men and women aged 30 to 64 years in France. Results of two national surveys. Ann Readapt Med Phys. 2007;50: 640–644. 10.1016/j.annrmp.2007.05.009 17631977

[26] Hussain SM, Urquhart DM, Wang Y, Dunstan D, Shaw JE, Magliano DJ, et al Associations between television viewing and physical activity and low back pain in community-based adults: A cohort study. Medicine (Baltimore). 2016;95:e3963. 27336896 10.1097/MD.0000000000003963 PMC4998334

[27] Bener A, Dafeeah EE, Alnaqbi K. Prevalence and correlates of low back pain in primary care: what are the contributing factors in a rapidly developing country. Asian Spine J. 2014;8: 227–236. 10.4184/asj.2014.8.3.227 24967035 PMC4068841

[28] Taylor JB, Goode AP, George SZ, Cook CE. Incidence and risk factors for first-time incident low back pain: a systematic review and meta-analysis. Spine J.2014;14: 2299–319. 10.1016/j.spinee.2014.01.026 24462537

[29] Koes BW, van Tulder MW, Thomas S. Diagnosis and treatment of low back pain. BMJ. 2006;17;332: 1430–1434. 10.1136/bmj.332.7555.1430 16777886 PMC1479671

[30] Santos-Eggimann B, Wietlisbach V, Rickenbach M, Paccaud F, Gutzwiller F. One-year prevalence of low back pain in two Swiss regions: estimates from the population participating in the 1992–1993 MONICA project. Spine (Phila Pa 1976). 2000;25: 2473–2479. 11013499 10.1097/00007632-200010010-00009

[31] Heneweer H, Staes F, Aufdemkampe G, van Rijn M, Vanhees L. Physical activity and low back pain: a systematic review of recent literature. Eur Spine J. 2011;20: 826–845. 10.1007/s00586-010-1680-7 21221663 PMC3099170

[32] van Middelkoop M, Rubinstein SM, Verhagen AP, Ostelo RW, Koes BW, van Tulder MW. Exercise therapy for chronic nonspecific low-back pain. Best Pract Res Clin Rheumatol. 2010;24: 193–204. 10.1016/j.berh.2010.01.002 20227641

[33] Kamper SJ, Apeldoorn AT, Chiarotto A, Smeets RJ, Ostelo RW, Guzman J, et al Multidisciplinary biopsychosocial rehabilitation for chronic low back pain. Cochrane Database Syst Rev. 2014;9: CD000963. 10.1002/14651858.CD000963.pub3 PMC10945502 25180773

[34] Izzo R, Popolizio T, D'Aprile P, Muto M. Spinal pain. Eur J Radiol. 2015;84: 746–756. 10.1016/j.ejrad.2015.01.018 25824642

[35] Großschädl F, Stolz E, Mayerl H, Rásky É, Freidl W, Stronegger W. Educational inequality as a predictor of rising back pain prevalence in Austria-sex differences. Eur J Public Health. 2016;26: 248–253. 10.1093/eurpub/ckv163 26370439

[36] Murphy S, Blake C, Power CK, Fullen BM. Outcomes of a group education/exercise intervention in a population of patients with non-specific low back pain: a 3-year review. Ir J Med Sci. 2014; 183: 341–350. 10.1007/s11845-013-1013-z 24037101

[37] Gomes JL, Kingma M, Kamper SJ, Maher CG, Ferreira PH, Marques AP, et al The association between symptom severity and physical activity participation in people seeking care for acute low back pain. Eur Spine J. 2015;24: 452–457. 10.1007/s00586-015-3763-y 25597041

[38] Krismer M, van Tulder M. Strategies for prevention and management of musculoskeletal conditions. Low back pain (non-specific). Best Pract Res Clin Rheumatol. 2007;21: 77–91. 17350545 10.1016/j.berh.2006.08.004

[39] Ramond-Roquin A, Bouton C, Bègue C, Petit A, Roquelaure Y, Huez JF. Psychosocial risk factors, interventions, and comorbidity in patients with non-specific low back pain in primary care: need for comprehensive and patient-centered care. Front Med (Lausanne). 2015; 2: 73. 26501062 10.3389/fmed.2015.00073 PMC4597113

[40] Schaller A, Dejonghe L, Haastert B, Froboese I. Physical activity and health-related quality of life in chronic low back pain patients: a cross-sectional study. BMC Musculoskelet Disord. 2015;16: 62 10.1186/s12891-015-0527-0 25887138 PMC4373082

[41] Ribaud A, Tavares I, Viollet E, Julia M, Hérisson C, Dupeyron A. Which physical activities and sports can be recommended to chronic low back pain patients after rehabilitation? Ann Phys Rehabil Med. 2013;56: 576–594. 10.1016/j.rehab.2013.08.007 24140440

[42] Hendrick P, Milosavljevic S, Hale L, Hurley DA, McDonough S, Ryan B, et al The relationship between physical activity and low back pain outcomes: a systematic review of observational studies. Eur Spine J. 2011;20: 464–474. 10.1007/s00586-010-1616-2 21053026 PMC3048226

[43] Hayden JA, van Tulder MW, Malmivaara A, Koes BW. Exercise therapy for treatment of non-specific low back pain. Cochrane Database Syst Rev. 2005; 3: CD000335. 10.1002/14651858.CD000335.pub2 PMC10068907 16034851

[44] Jang JH, Cho TY, Cho YH. The effects of t'ai chi on muscle activity, pain, and balance in females in their 20s with acute low back pain. J Phys Ther Sci. 2015;27: 725–727. 10.1589/jpts.27.725 25931717 PMC4395701

[45] Garber CE, Blissmer B, Deschenes MR, Franklin BA, Lamonte MJ, Lee IM, et al American College of Sports Medicine position stand. Quantity and quality of exercise for developing and maintaining cardiorespiratory, musculoskeletal, and neuromotor fitness in apparently healthy adults: guidance for prescribing exercise. Med Sci Sports Exerc. 2011;43:1334–59. 21694556 10.1249/MSS.0b013e318213fefb

